# Risk of violence from the man involved in the pregnancy after receiving or being denied an abortion

**DOI:** 10.1186/s12916-014-0144-z

**Published:** 2014-09-29

**Authors:** Sarah CM Roberts, M Antonia Biggs, Karuna S Chibber, Heather Gould, Corinne H Rocca, Diana Greene Foster

**Affiliations:** Advancing New Standards in Reproductive Health (ANSIRH), University of California, San Francisco, 1330 Broadway, Suite 1100, Oakland, CA 94612 USA

**Keywords:** Abortion, Intimate partner violence

## Abstract

**Background:**

Intimate partner violence is common among women having abortions, with between 6% and 22% reporting recent violence from an intimate partner. Concern about violence is a reason some pregnant women decide to terminate their pregnancies. Whether risk of violence decreases after having an abortion, remains unknown.

**Methods:**

Data are from the Turnaway Study, a prospective cohort study of women seeking abortions at 30 facilities across the U.S. Participants included women who: presented just prior to a facility’s gestational age limit and received abortions (Near Limit Abortion Group, n = 452), presented just beyond the gestational limit and were denied abortions (Turnaways, n = 231), and received first trimester abortions (First Trimester Abortion Group, n = 273). Mixed effects logistic regression was used to assess the relationship between receiving versus being denied abortion and subsequent violence from the man involved in the pregnancy over 2.5 years.

**Results:**

Physical violence decreased for Near Limits (adjusted odds ratios (aOR), 0.93 per month; 95% Confidence Interval (CI) 0.90, 0.96), but not Turnaways who gave birth (*P* < .05 versus Near Limits). The decrease for First Trimesters was similar to Near Limits (*P* = .324). Psychological violence decreased for all groups (aOR, 0.97; CI 0.94, 1.00), with no differential change across groups.

**Conclusions:**

Policies restricting abortion provision may result in more women being unable to terminate unwanted pregnancies, potentially keeping them in contact with violent partners, and putting women and their children at risk.

## Background

Experiencing violence, especially from intimate partners, is common among women having abortions, with 6% to 22% reporting recent violence from an intimate partner [1-5]. Concern about violence is a reason some pregnant women decide to terminate their pregnancies [6-9]. In particular, women who report violence as a reason for abortion describe not wanting to expose children to violence and believing that having the baby will tether them to an abusive partner [6].

Whether having an abortion actually allows women to evade intimate partner violence (IPV) remains unknown. One prospective study in New Zealand found elevated levels of past year IPV among women who had abortions compared to women who gave birth and no differences between women who had abortions and women who had not been pregnant [10]. However, the difference in IPV between women who had an abortion and women who gave birth was no longer statistically significant once confounders were controlled. The New Zealand study assessed IPV from any intimate partner, not necessarily from the man involved in the pregnancy (MIP). Focusing on the MIP is important because this is the person to whom a woman would be linked if she carried the pregnancy to term.

The aim of this paper is to examine changes in violence from the MIP among women receiving versus being denied abortion over 2.5 years after seeking an abortion. Comparing changes in violence over time between women receiving versus denied abortion has the benefit of being able to better match groups of women with respect to important confounding factors, such as pregnancy intentions and violence, that can lead women to become pregnant and also decide to terminate a pregnancy. Thus, women denied abortions better represent what women’s experiences would have been had they not terminated the unwanted pregnancy and allow for the possibility of causal inference regarding outcomes subsequent to abortion.

## Methods

Data for this paper come from the Turnaway Study, a prospective cohort study of women who all sought, but did not all receive, abortions at 30 abortion facilities in the United States. Women were recruited when they sought abortion and were interviewed by telephone one week later. The Turnaway Study is following participants for five years, interviewing them by telephone biannually. This paper presents findings from the first 2.5 years of data collection. The University of California, San Francisco’s Committee for Human Research granted ethical approval for the study. All participants provided written informed consent.

Participants were English- and Spanish-speaking women with no known fetal anomalies or demise, 15-years-old or older, presenting at one of the study facilities between January 2008 and December 2010 (the recruitment period). Facilities with the latest gestational age limit for providing abortion within 150 miles were eligible. All but two facilities approached participated; one was replaced with a facility with a similar catchment area, identical gestational limit and similar patient volume. Participating facility limits ranged from 10 weeks through the end of the second trimester, with four having limits in the first trimester, eight between 14 and less than 20 weeks, and 18 after 20 weeks. Details about the study and facilities have been published previously [11-16].

Women were eligible for the study and assigned to one of three study groups based on their gestational age at abortion-seeking. Women presenting for abortion within two weeks under a facility’s gestational age limit and receiving an abortion were assigned to the Near Limit Abortion Group*;* women presenting for abortion up to three weeks over the limit and denied abortion at that facility were assigned to the Turnaway Group*.* Near Limit Abortion versus Turnaway is the main comparison in this study. For every Turnaway, we recruited two women for the Near Limit Abortion Group and also one woman receiving an abortion in the first trimester for the First Trimester Abortion Group. The First Trimester Abortion Group was included to assess how the experiences of women in the Near Limit Group compared to the more typical experience of abortion in the U.S., where 90% of abortions occur in the first trimester [17].

Of eligible participants approached, 37.5% consented, with 85% of those consenting (n = 956) completing the baseline interview [16]. Seventy two percent of those completing the baseline interview were retained at the sixth interview (2.5 years). There was no differential participation across the two main study groups (Near Limit Abortion Group and Turnaway Group), but fewer women eligible for the First Trimester Abortion Group participated. There was no differential loss to follow up by study group or by baseline violence over the 2.5 years. Of the 956 who completed a baseline interview, 452 were in the Near Limit Abortion Group, 231 in the Turnaway Group, and 273 in the First Trimester Abortion Group. Some women in the Turnaway Group received an abortion elsewhere or miscarried subsequent to being denied the abortion at the recruitment facility. At one facility with a gestational limit of 10 weeks, 90% of Turnaways received an abortion elsewhere or miscarried. All of the 76 participants from this facility were excluded from analyses. Two Near Limit Abortion Group and one First Trimester Group participants later reported that they had not had the abortion and were excluded from the analyses. Women who did not know who the man involved in the pregnancy was or reported that the pregnancy was a result of rape (n = 15) were not asked questions about the MIP at follow-up interviews and were excluded from the analyses. The sample thus includes 862 participants, with 405 in the Near Limit Abortion Group, 156 Turnaways who had a birth (Turnaway Births), 48 Turnaways who had an abortion (n = 43) or miscarriage (n = 5) (Turnaway No Births), and 253 in the First Trimester Abortion Group.

Two types of violence from the MIP were considered as outcome variables: physical and psychological. These outcome variables were based on questions about physical violence (that is, ‘pushed, hit, slapped, kicked, choked, or physically hurt in any way by another person’) and psychological violence (that is, ‘frightened for your safety as a result of anger or threats made by another person’) in the last six months. We also asked whether the perpetrator of the most recent violent episode was the MIP. The violence questions were asked at each biannual interview. Violence questions were modified from California’s Maternal and Infant Health Assessment Survey [18]. During the baseline interview, participants were also asked about physical and psychological violence in the year preceding the interview. We used dates of conception and of violence to determine whether the violence occurred during or before pregnancy.

Study group was the main independent variable and included Near Limit Abortion Group (as reference); Turnaway Births (Turnaways with a live birth, including 15 who placed their baby for adoption); Turnaway No Births (Turnaways who received an abortion elsewhere or miscarried) and First Trimester Abortion Group. Because we were interested in both Near Limit versus Turnaway Birth and Near Limit versus First Trimester comparisons, Near Limit as the reference allowed simultaneous comparisons of both sets of study groups. Months was the time variable and was measured in months since recruitment. Study Group X Months interaction terms allowed examination of group-specific change over time.

Covariables included potential confounders of the relationship between study group and subsequent violence, all measured at baseline. We measured race/ethnicity (White, Black, Hispanic/Latina, Other); age in years; employment (employed full or part time versus unemployed); union status (married, cohabiting, not-cohabiting never married, divorced/widowed); history of child abuse/neglect, or reporting having ever experienced physical abuse, neglect, or sexual abuse during childhood; raising children (no live births, living with all biological children, one or more biological children cared for by someone else); grew up in a household with someone with an alcohol or drug problem; grew up in a household with someone with a psychological disorder; previous depression or anxiety diagnosis; alcohol problem symptom the month before pregnancy recognition, with those reporting having a drink first thing in the morning to steady nerves or get rid of a hangover and those reporting that they were unable to remember what happened the night before because of drinking coded as having a problem symptom; and illicit drug use the month before pregnancy recognition, with any marijuana, heroin, cocaine, methamphetamine use or prescription drug misuse coded as illicit drug use.

We used three analytical approaches. First, we used mixed effects linear, logistic and multinomial logistic regression to compare baseline characteristics of study groups. The mixed effects regression models included random intercepts for facility to account for clustering of participants by site. Second, longitudinal analyses examining associations between study group and violence over time were conducted with mixed effects multivariate logistic regression. Longitudinal analyses included data from the first six interviews, from one week through 2.5 years after abortion-seeking, and included all available data. Random intercepts for facility and for individual were included to account for facility and individual-level clustering. Random slopes for individual did not improve any model fits and, thus, were not included. Third, in the cases where change over time differed between the Near Limit and Turnaway Births groups, a model with Turnaway Births as the reference group was estimated to be able to describe change over time directly for the Turnaway Births group and not describe change solely in relation to the Near Limit Group. All analyses were conducted in Stata 12.0.

## Results

Participant baseline characteristics are shown in Table 1. Compared to the Near Limit Abortion Group, the Turnaway Birth Group was younger, less likely to be employed and less likely to be raising children. A smaller proportion of women in the Turnaway No Birth Group than in the Near Limit Abortion Group reported a history of child abuse/neglect and growing up in a household with someone with a drinking or drug problem. Compared to the Near Limit Abortion Group, the First Trimester Abortion Group was more likely to be White, employed and report growing up in a household with someone with a psychological disorder. As expected due to study design, groups differed significantly on gestational age at recruitment.

**Table 1:** **Baseline characteristics across study group**

**Participant characteristic**	**Total**	**Near limit abortion**	**Turnaway births**	**Turnaway no births**	**First -trimester abortion**
	**number = 862**	**number = 405**	**number = 156**	**number = 48**	**number = 253**
	**mean (SD) or %**				
Age, years	24.9 (5.8)	25.0(5.9)	23.5(5.6) **	24.5(6.3)	25.8(5.7) +
Race/ethnicity					*
White	33	32	25	44	39
Black	32	31	35	27	32
Hispanic/Latina	22	21	28	13	21
Other	13	16	13	17	8
Employed	54	55	40 **	50	64 *
Gestational age, weeks	16.9 (7.0)	19.9 (4.0)	23.3 (3.4) ***	18.9 (3.9) ***	7.6 (2.3) ***
Union status					
Married	9	8	10	6	11
Cohabiting	18	18	13	17	21
Not-cohabiting, never married	63	63	72	61	57
Divorced/widowed	10	11	5	17	11
Raising children			*		
No live births	38	34	47	42	38
Living with all biological children	53	56	42	52	55
Has 1+ children cared for by someone else	9	9	11	6	7
History of child abuse/neglect	26	26	26	13*	27
Family history before age 18					
Household member drinking/drug problem	20	21	15	6*	25
Household member psychological disorder	11	8	8	6	18***
Previous depression or anxiety diagnosis	25	23	20	29	30
Alcohol problem symptom	6	4	7	10	7
Recent drug use	14	13	13	8	18
Physical violence from MIP prior six months	5	6	3	4	4
Psychological violence from MIP prior six months	3	3	3	4	4

+*P* < .10, *P < .05, **P < .01, ***P < .001. MIP, man involved in the pregnancy.

In the six months prior to baseline (to match the timeframe for subsequent measures of violence), 5% of the participants experienced physical violence from the MIP and 3% reported psychological violence from the MIP (See Table 1). There were no statistically significant differences across study group in either physical or psychological violence from the MIP in the six months prior to baseline.

**Figure 1:**
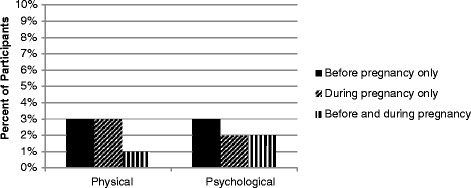
**Violence from the man involved in the pregnancy over the past year, baseline.**

In the year prior to baseline, violence from the MIP occurred both before and during pregnancy, with about 3% reporting physical violence before pregnancy only, 3% during pregnancy only and 1% both before and during pregnancy (Figure 1, not shown in a table). For psychological violence from the MIP, this was 3% before pregnancy only, 2% during pregnancy only and 2% both before and during pregnancy.

**Figure 2:**
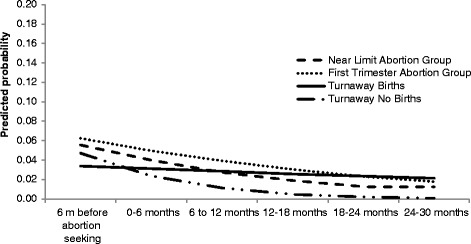
**Physical violence from the man involved in the pregnancy.**

Results of longitudinal analyses are presented in Table 2 and are shown graphically in Figure 2. The adjusted odds ratio (aOR) for Study Group indicates the extent to which violence at baseline for each study group differed from the Near Limit Abortion Group. Months indicates change over time in violence for the Near Limit Abortion Group and the *P* value for months indicates whether the slope of change over time statistically differed from zero. *P* values for Study Group X Time interactions indicate whether change over time differed for that study group versus change over time for the Near Limit Abortion group.

**Table 2:** **Multivariate mixed effects logistic regressions of physical and psychological violence from the man involved in the pregnancy over 2.5 years**

**Physical violence (number = 848)**				
	**aOR**	***P*** **value**	**95% CI**	
**Study Group**				
Near Limit Abortion Group	ref			
Turnaway Births	0.58	0.288	0.22	1.57
Turnaway No Birth	1.04	0.963	0.20	5.42
First Trimester Abortion Group	1.05	0.895	0.50	2.22
**Time**				
Months	0.93	<0.001	0.90	0.96
**Study Group X Time interactions**				
Turnaway Births X months	1.06	0.047	1.00	1.12
Turnaway No Birth X months	0.93	0.406	0.78	1.11
First Trimester X months	1.02	0.324	0.98	1.07
**Psychological violence (number = 849)**				
	aOR	*P* value	95% CI	
**Study Group**				
Near Limit Abortion Group	ref			
Turnaway Births	0.97	0.962	0.33	2.85
Turnaway No Birth	2.47	0.290	0.46	13.14
First Trimester Abortion Group	1.28	0.564	0.56	2.91
**Time**				
Months	0.97	0.043	0.94	1.00
**Study Group X Time interactions**				
Turnaway Births X months	1.02	0.451	0.97	1.08
Turnaway No Birth X months	0.83	0.126	0.66	1.05
First Trimester X months	1.02	0.394	0.98	1.06

aOR, adjusted odds ratio; CI, confidence interval.

Models adjust for: baseline age, race, employment, union status, raising children, depression/anxiety history, child abuse/neglect history, problem alcohol use prior to pregnancy recognition, recent drug use and having a household member with a drinking or drug problem or a psychiatric disorder during childhood.

There were no statistically significant differences in physical violence from the MIP in the six months prior to baseline across study groups. Physical violence from the MIP decreased over time for the Near Limit Group (aOR 0.93, *P* < .001) (See Table 2, Figure 2). Change in physical violence over time was similar in the Turnaway No Births and First Trimester groups as in the Near Limit group. However, change in physical violence differed over time between Turnaway Births and the Near Limit Abortion Group (aOR 0.98 for Turnaway Births, *P* = .047 compared to Near Limits). In a model with Turnaway Births as the reference group, the *P*-value for change over time was not significant (*P* = 0.396). This indicates that, unlike the other three groups, the Turnaway Births group did not experience a statistically significant decrease in physical violence from the MIP over time.

Psychological violence from the MIP decreased over time for the Near Limit Abortion Group (aOR = 0.97, *P* = .043) (See Table 2), with no differences at baseline or in change over time by study group; all groups experienced decreased psychological violence from the MIP.

## Discussion

Among women seeking abortion, having an abortion was associated with a reduction over time in physical violence from the MIP, while carrying the pregnancy to term was not. Terminating an unwanted pregnancy may allow women to avoid physical violence from the MIP, while having a baby from an unwanted pregnancy appears to result in sustained physical violence over time. This finding is consistent with our hypothesis that having a baby with an abusive man, compared to terminating the unwanted pregnancy, makes it harder to leave the abusive relationship. It is also consistent with findings from analyses of relationship outcomes among women in the Turnaway Study sample [19]. These analyses found that women denied abortions were slower to end their romantic relationships with the MIPs than women having abortions, with differences in romantic involvement disappearing by two years post-abortion seeking. They also found that women denied abortion were more likely to have sustained contact with the MIP over time. Notably, women having first-trimester abortions and women initially denied abortions who did not end up giving birth experienced similar reductions in violence as those having near-limit abortions.

IPV has serious health consequences for women, including injury, chronic pain, gastrointestinal problems, sexually-transmitted infections, depression and post-traumatic stress disorder [20]. The fact that women who had babies resulting from unwanted pregnancies had more ongoing physical violence is of particular concern, as the violence can also affect their children. Violence during pregnancy is associated with negative birth outcomes, including low birth weight, pre-term delivery and neonatal death [21], and children exposed to IPV are at increased risk of emotional and behavioral problems [22,23]. Ensuring that women unable to terminate unwanted pregnancies receive support and interventions around violence is of utmost importance; guidance is provided in a recent American College of Obstetricians and Gynecologists Opinion [24]. We found no differences in reports of violence prior to abortion seeking by study group; women who sought near-limit abortions did not report more violence than women who sought earlier abortions.

Some limitations are worth noting. First, women in the Near Limit group received abortions later in gestation than typical in the U.S. [17]; thus, it is unclear whether their experiences generalize to the more typical experience. However, when we compared women receiving abortions near gestational limits to women receiving first trimester abortions, we found no differential change over time. Second, violence from the MIP is based on who perpetrated the most recent episode of violence. If women were experiencing violence from multiple sources and the MIP was not the most recent source of violence, this may underestimate violence from the MIP. Third, information about violence from the MIP is based on self-reports by the woman experiencing the violence and may be under-reported. It is worth noting, though, that the proportion reporting violence from the MIP is in the range of estimates of past year intimate partner violence reported in other studies of abortion patients [2,3]. Fourth, the response rate is 37.5% and those who did not participate could differ from those who did participate on key characteristics. A recent review found that most prospective cohort studies published in high impact journals did not report participation information [25], meaning that published response rates for prospective cohort studies may suffer from reporting bias, with only those with higher rates reporting them [26]. The 37.5% response rate for a five year study with biannual interviews of women seeking a stigmatized health service is within the range of other large-scale prospective studies. Importantly, our exposure (receiving versus being denied abortion) was not associated with non-participation. Because the topic of violence was not raised when potential participants were initially informed about the study, non-participation is unlikely to be related to violence outcomes. Fifth, our violence measures did not capture severity or frequency of violence.

This study also has a number of strengths. First, the Turnaway Study is the first study of abortion and subsequent intimate partner violence to use a comparison group for women receiving abortions that best represents what women’s experiences would have been had they not been able to terminate their pregnancies. It is also a prospective study that was designed to assess experiences and health subsequent to abortion and, thus, does not rely on retrospective reports of abortion. Second, we found similar proportions experiencing violence from the MIP in the past year to previous studies of IPV among women having abortions, suggesting that the experiences of women in our sample may be typical of those of other women seeking abortion [2,3]. Third, retention was high, with 72% of participants retained at the sixth interview and no differential loss to follow-up by study group.

## Conclusions

In summary, physical violence from the MIP decreased over time for women having abortions but not for women denied abortions. This finding is concerning, especially in light of the increasing number of state-based restrictions that limit women’s access to abortion care in the U.S. Policies that restrict abortion provision may result in more women being unable to terminate unwanted pregnancies, potentially keeping some women in physically violent relationships, and putting both women and their children at increased risk of violence and other negative health consequences.
